# Short-chain fatty acid receptors inhibit invasive phenotypes in breast cancer cells

**DOI:** 10.1371/journal.pone.0186334

**Published:** 2017-10-19

**Authors:** Madhumathi Thirunavukkarasan, Chao Wang, Angad Rao, Tatsuma Hind, Yuan Ru Teo, Abrar Al-Mahmood Siddiquee, Mohamed Ally Ibrahim Goghari, Alan Prem Kumar, Deron R. Herr

**Affiliations:** 1 Department of Pharmacology, Yong Loo Lin School of Medicine, National University of Singapore, Singapore; 2 Cancer Science Institute of Singapore, National University of Singapore, Singapore, Singapore; 3 Department of Pharmacology, University of British Columbia, Vancouver, BC, Canada; 4 Curtin Health Innovation Research Institute, Curtin University, Perth, Western Australia, Australia; 5 National University Cancer Institute, Singapore, Singapore; 6 Department of Biological Sciences, University of North Texas, Denton, TX, United States of America; 7 Department of Biology, San Diego State University, San Diego, CA, United States of America; University of South Alabama Mitchell Cancer Institute, UNITED STATES

## Abstract

Short chain fatty acids (2 to 6 carbons in length) are ubiquitous lipids that are present in human plasma at micromolar concentrations. In addition to serving as metabolic precursors for lipid and carbohydrate synthesis, they also act as cognate ligands for two known G protein-coupled receptors (GPCRs), FFAR2 and FFAR3. While there is evidence that these receptors may inhibit the progression of colorectal cancer, their roles in breast cancer cells are largely unknown. We evaluated the effects of enforced overexpression of these receptors in two phenotypically distinct breast cancer cell lines: MCF7 and MDA-MD-231. Our results demonstrate that both receptors inhibit cell invasiveness, but through different signaling processes. In invasive, mesenchymal-like MDA-MB-231 cells, FFAR2 inhibits the Hippo-Yap pathway and increases expression of adhesion protein E-cadherin, while FFAR3 inhibits MAPK signaling. Both receptors have the net effect of reducing actin polymerization and invasion of cells through a Matrigel matrix. These effects were absent in the less invasive, epithelial-like MCF7 cells. Correspondingly, there is reduced expression of both receptors in invasive breast carcinoma and in aggressive triple-negative breast tumors, relative to normal breast tissue. Cumulatively, our data suggest that the activation of cognate receptors by short chain fatty acids drives breast cancer cells toward a non-invasive phenotype and therefore may inhibit metastasis.

## Introduction

Metastasis is a multi-stage process involving genetically unstable tumor cells that undergo phenotypic changes that allow them to egress from the primary tumor and colonize a distant tissue site with a favorable microenvironment [1, 2]. Metastasis is responsible for 90% of cancer mortality, yet its molecular processes remain incompletely characterized [3]. One of the essential mechanisms involved in metastasis is epithelial-mesenchymal transition (EMT). EMT is a key process that governs normal physiological events like embryogenesis, morphogenesis, and wound healing. However, EMT in cancer progression involves differentiation of adherent epithelial-like tumor cells to potentially pluripotent cells of a migratory and invasive mesenchymal phenotype [4]. One of the characteristic features of EMT is loss of E-cadherin expression, which leads to loss of ability to maintain cell-cell adhesion, integrity and is collaterally linked to migratory and invasive properties of cancer cells [5]. The phenotype alteration involved in EMT is regulated by multiple transcription factors: SNAIL, SLUG, TWIST, and ZEB [6–8]. Moreover, there are a number of growth factors (e.g. transforming growth factor β, epidermal growth factor, platelet-derived growth factor, and fibroblast growth factor) [7–10], intracellular signal transducing proteins (e.g. extracellular signal–regulated kinase, mitogen-activated protein kinase, phosphatidylinositol-3-kinase, and protein kinase B), and transcriptional cofactors (e.g. yes-associated protein 1 (YAP1), Kirsten rat sarcoma viral oncogene homolog) [11], that have been identified as stromal-derived factors that mediate EMT-inducing signals. More recently, studies have implicated specific G protein-coupled receptors (GPCRs) as important mediators of EMT-related processes [12]. These include CXCR4, CCR7, PAR1 [13, 14], and receptors with bioactive lipid ligands, such as S1P_1,3_ [15–17], LPA_1-6_ [18, 19], and prostaglandin receptors (EP_1_ & EP_4_) [20, 21]. The discovery of free fatty acid receptors (FFARs) in the past decade introduced a new paradigm of lipid-mediated regulation of cellular processes: free fatty acids as extracellular signaling molecules [22, 23]. The FFAR family is composed of 4 *bona fide* members: FFAR1 (GPR40), FFAR2 (GPR43), FFAR3 (GPR41) and FFAR4 (GPR120). Among these, FFAR1 and FFAR4 are activated by long and medium chain fatty acids (LCFA/MCFA) such as oleate and palmitate, while FFAR2 and FFAR3 are activated by short chain fatty acids (SCFA) such as propionate and butyrate [22–25]. FFARs are known to participate in a wide range of physiological functions, such as regulation of inflammatory mediators [26], leptin production [26], production of peptide YY [27], glucose-stimulated-insulin secretion [28], and secretion of glucagon-like peptide [29]. Similarly, recent data also suggest that FFAR2 and FFAR3 play a role in tumor suppression. FFAR2 has been reported to have significantly reduced expression in colorectal adenocarcinoma, and propionate, the ligand for FFAR2 and FFAR3, is shown to reduce proliferation and promote apoptosis in several cancer cell types [26, 30–33]. The short chain fatty acid butyrate is also reported to inhibit growth and proliferation by inhibiting HDAC activity and inducing apoptosis [31, 34]. Taken together these studies support a model by which reduced expression of FFAR2 and FFAR3 may contribute to cancer progression.

Previous studies suggest that FFAR2 and FFAR3 are involved in the regulation of cancer development. This led us to investigate the mechanisms underlying FFAR2- and FFAR3-mediated effects on breast cancer cells, particularly pertaining to metastasis. In this study, we report that expression of FFAR2 and FFAR3 regulates YAP1 phosphorylation and that FFAR2 also regulates E-cadherin in MDA-MB-231 cells. Furthermore, through siRNA knockdown studies we identify LATS1, an upstream regulator of YAP1, as an obligate mediator of the E-cadherin changes observed in FFAR2 expressing MDA-MB-231 cells. Unlike FFAR2, FFAR3-mediated YAP1 elevation does not correlate with E-cadherin levels, which is likely to be regulated instead by the MAPK/ERK1/2 pathway.

## Materials and methods

### Antibodies and reagents

Primary antibodies: E-cadherin (Cell Signaling Technologies #3195), total YAP1 (Santa Cruz #G2710), Phospho-YAP1 S127 (Cell Signaling Technologies #4911S), LATS1 (Cell Signaling Technologies #3477S), p44/42 MAPK (Cell Signaling Technologies #9102), Phospho-p44/42 MAPK (Cell Signaling Technologies #9101), β-actin antibody (Sigma #A 5316), and V5 tag antibody (ThermoFisher Scientific #R960-25). Secondary antibodies: Horseradish peroxidase-conjugated goat anti-rabbit (Cell Signaling Technologies #7074) and anti-mouse (Cell Signaling Technologies #7076), Alexa Fluor 594 (ThermoFisher Scientific #A11005) and Alexa Fluor 488 phalloidin (ThermoFisher Scientific #A12379).

Reagents: Propionate (propionic acid # 402907) from Sigma-Aldrich Corporation.

### Cell culture

MDA-MB-231 (ATCC #HTB-26), MCF-7 cells (ATCC #HTB-22), HEK293 cells (ATCC #CRL-1573) and HEK293T cells (ATCC #CRL-3216) were maintained as monolayer cultures on tissue culture dishes at 37°C, 5% CO2, 100% humidity in Dulbecco’s modified Eagle’s medium (DMEM) supplemented with 10% heat-inactivated fetal bovine serum (FBS)and antibiotics.

### Plasmid constructs and generation of stable cell lines

FFAR2-V5 and FFAR3-V5 were constructed by amplifying the FFAR2/FFAR3 coding DNA sequence (CDS) utilizing FFAR2 forward primer 5’-TGGGTCTGGTCTTTGGGTTG-3’ and the reverse primer 5’-CACGTAGCAGAAGGCTGTGA-3’ and FFAR3 forward primer 5’- AACCTCACCCTCTCGGATCT and reverse primer 5’- GCCGAGTCTTGTACCAAAGC, the amplified product was inserted into pcDNA3.1/V5-His TOPO expression vector according to manufacturer’s instructions.

To generate MCF-7 and MDA-MB-231 cell lines stably overexpressing either empty vector pcDNA3.1/V5-His (referred to as vector control) or the FFAR2-V5 and FFAR3-V5 (referred to as FFAR2- and FFAR3-overexpressing), respective plasmid constructs were linearized with restriction enzyme PvuI-HF following which they were transfected into the cells with Lipofectamine 3000 (Thermo Fisher #L3000015). The transfected cells were cultured in 1 mg/ml G418 to select for stable transfectants.

For LATS1 knockdown studies, the indicated cells were transfected with 75 pmol of control (5’-UUCUCCGAACGUGUCACGU-3’) or target-specific (5’-CAUACGAGUCAAUCAGUAA-3’) siRNA using Lipofectamine 3000 reagent per manufacturer’s instructions. Cells were incubated for 48 hours after transfection, and processed for western analysis.

### Western blot

Western analysis was performed essentially as described [35]. Cell lysate was prepared by treating the cells grown on 6-well tissue culture plates at varying time points for different experiments under starvation condition in serum-free DMEM. The cells were washed in ice cold phosphate-buffered saline (PBS) and then incubated with lysis buffer (1% Triton X-100/ 250 mM NaCL / 0.5 mM EGTA (Ethylene glycol tetraacetic acid) / 2 mM EDTA (Ethylenediaminetetraacetic acid) / 20 mM HEPES) containing protease and phosphatase inhibitors cocktail for 20 minutes on ice and harvested with a cell scraper. The cell suspension was centrifuged at 15,000 x g for 15 minutes at 4°C. Protein concentration was quantified using a Bio-Rad protein assay dye (Bio-Rad #500006) and three parts of sample was diluted to one part of SDS-loading dye and denatured at 98°C for 10 minutes. 20–25 μg protein was separated on a 10% SDS-polyacrylamide gel, transferred to PVDF membrane and blocked for 1 hour at room temperature in 5% nonfat milk in Tris-buffered saline containing Tween 20 (TBS-T). The blot was then incubated overnight with primary antibody at 1:2,000 dilution for anti-E-Cadherin, anti-YAP1, anti-ERK and at 1:10,000 for anti- β-actin; washed three times with TBS-T for 5 minutes each and incubated with horseradish peroxidase conjugated secondary antibody (1:10,000) for 2 hours; and visualized using the WesternBright ECL HRP substrate (Advansta, K-12045-D20). Image J software was used to quantify the images.

### Immunoctochemistry

Immunocytochemistry was performed essentially as described [36]. Cells were cultured on collagen coated coverslips, fixed with 4% paraformaldehyde for 15 minutes, washed four times in 1x PBS, blocked for 1 hour in blocking solution (PBS/2.5% BSA/0.3% Triton X-100) at room temperature, incubated overnight with unconjugated primary antibodies in 10% blocking buffer, and washed with PBS. Coverslips were then incubated with secondary antibodies conjugated to Alexa Fluor dyes (Invitrogen) at 1 μg/ml in blocking solution for 1 hour with gentle rocking, then washed, incubated with 4',6-diamidino-2-phenylindole (DAPI) at 500 nM for 10 minutes, and then mounted on glass slides with Vectashield (Vector laboratories).

### Invasion assay

The *in vitro* invasion assay was performed essentially as described [37]. Briefly, MDA-MB-231 (8 × 10^4^) cells were seeded onto the top chamber of a Matrigel invasion chamber (Corning #354480) in serum-free media and the lower part of the chamber was in contact with 10% FBS containing culture media. The treatment where mentioned was carried out by adding 1 mM propionate diluted in plain DMEM to the upper chamber and culture media with 10% FBS was added to the lower chamber. Following a 24-hour incubation, the upper surfaces of the transwell chambers were gently wiped with cotton swabs and the invading cells were fixed in methanol, stained with crystal violet solution, washed, and air dried. The invading cell numbers were counted in five randomly selected microscope fields (20×).

### Quantitative reverse transcription PCR (qPCR)

mRNA quantification was performed essentially as described [38]. Total RNA was isolated using Trizol reagent (Thermo Scientific) per manufacturer’s instructions. Approximately 1 μg of each sample was primed with oligo(dT)18 and random hexamer primers prior using Maxima First Strand cDNA synthesis kit (Thermo Scientific). For quantitative real-time RT-PCR, targets were amplified with Maxima SYBR Green/ROX qPCR Master Mix (Thermo Scientific) on an Applied Biosystems ViiA 7 Real-Time PCR System (Thermo Scientific) using gene-specific primer pairs. Relative gene expression was determined using the 2^-ΔΔCT^ method as described [39].

### TGFα shedding assay

This assay was conducted essentially as described [40, 41]. Briefly, HEK293 cells were co-transfected with expression constructs for alkaline phosphatase-TGFα and the indicated receptor, along with chimeric G protein (Gq/i) using Lipofectamine 3000. Following a 24-hour incubation they were collected by trypsinization, washed with 1x PBS, and seeded into 96-well plates in Hank’s balanced saline solution (HBSS). Stimulation was carried out for 1 hour following which alkaline phosphatase activity was detected in both cells and supernatant. Activity of the receptor (% shedding) was described as the alkaline phosphatase activity of the supernatant/ divided by the total alkaline phosphatase activity (cells + supernatant). Data processing and statistical analyses were performed with GraphPad Prism 6.

## Results

### FFAR2 and FFAR3 differentially promote an epithelial phenotype

To understand the functions of FFAR2 and FFAR3 in breast cancer metastasis, we evaluated two breast cancer cell lines that have contrasting EMT phenotypes reflected by their EMT score: 1.0 = highly mesenchymal, -1.0 = highly epithelial [42]. MCF-7 cells are epithelial-like (EMT score = -0.412) and have high E-cadherin expression, while MDA-MB-231 cells are mesenchymal-like (EMT score = 0.454) and have low E-cadherin expression. The selected cell lines were stably transfected with V5-tagged FFAR2, FFAR3, or pcDNA3.1 vector control. The receptor over-expression was validated by immunocytochemistry and visualization with an anti-V5 antibody (S1 Fig).

**Fig 1:**
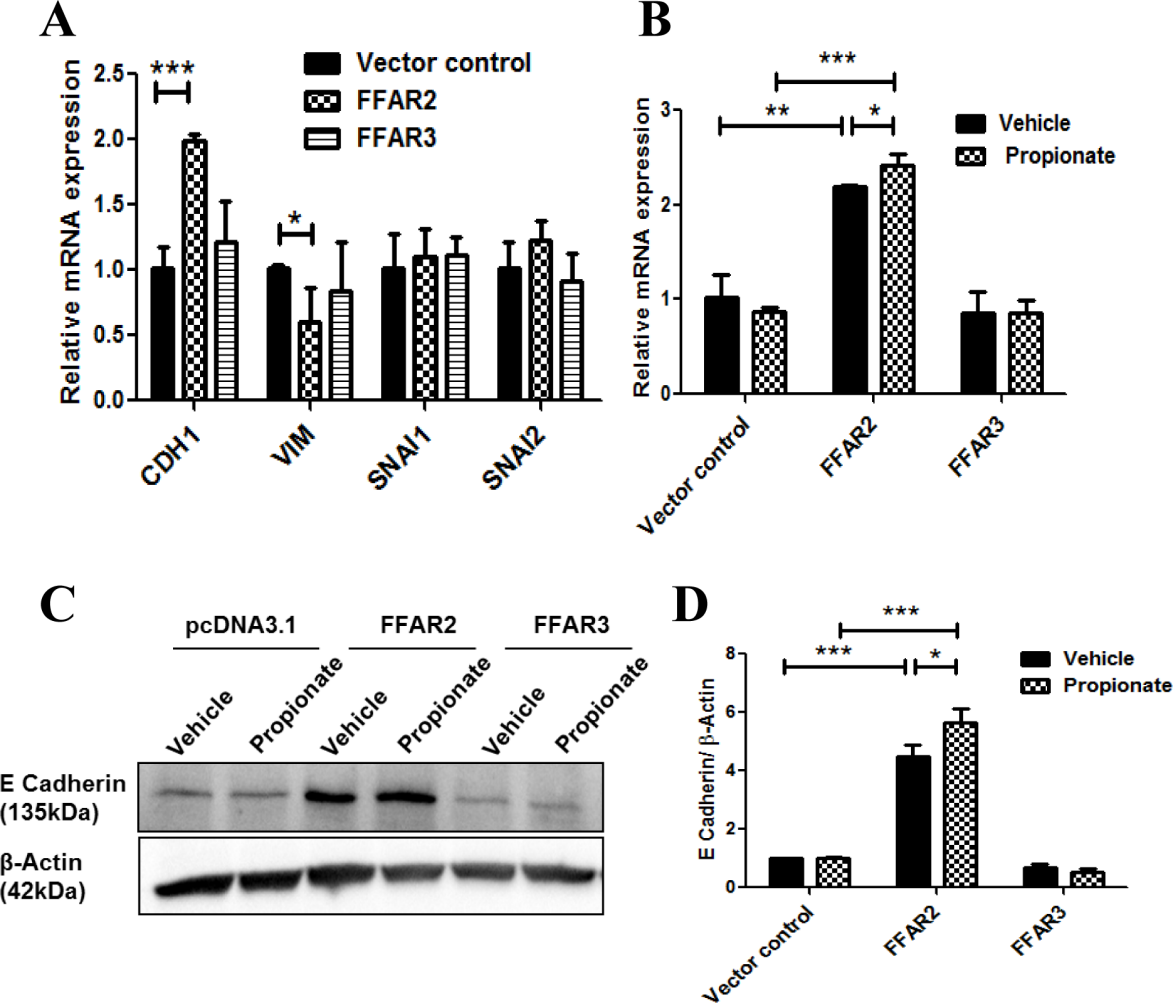
FFAR2 regulates E-cadherin. (A) qRT-PCR analysis of EMT-related genes was performed on MDA-MB-231 cells overexpressing FFAR2 and FFAR3. Expression of *CDH1* was selectively increased in FFAR2-overexpressing cells relative to vector controls, while no changes in EMT-related genes were observed in FFAR3-overexpressing cells (N = 3). (B) Cells were treated with vehicle control or with 100 μM propionate for 24 hours, then evaluated for *CDH1* expression by qRT-PCR. FFAR2-overexpressing cells, but not in vector control cells or in FFAR3-overexpressing cells, demonstrated a ligand-dependent increase in *CDH1* mRNA (N = 3). (C) Cells were treated with vehicle control or with 100 μM propionate for 24 hours, then evaluated for E-cadherin levels by western blot analysis. Results are representative of three independent experiments. (D) Quantitation of the relative density of the data from (C) demonstrates that E-cadherin is increased in FFAR2-overexpressing cells, and is further increased by propionate treatment (N = 3). (*p< 0.05, **p< 0.01, ***p< 0.001.).

As studies suggest that FFAR2 expression is reduced in certain cancers [30], and that proliferation may be inhibited by short chain fatty acids, butyrate and propionate [31, 43], we speculated that short chain fatty acid receptors might play a role in regulating breast cancer progression, and possibly metastasis. To evaluate EMT in our engineered cell lines, we carried out a limited qRT-PCR screen to investigate the expression levels of the critical genes known to be regulators of EMT (Fig 1A). *CDH1*, the gene encoding E-cadherin, was significantly upregulated in FFAR2-expressing cells relative to control cells and FFAR3-expressing cells (Fig 1A). Furthermore, stimulation of cells with propionate, a potent and endogenous ligand for both FFAR2 and FFAR3 (S2 Fig) [44], resulted in a further increase in E-cadherin expression in FFAR2-expressing cells (Fig 1B). The increased mRNA expression of E-cadherin corresponded with increased protein expression (Fig 1C and 1D). However, FFAR2-expressing MCF-7 cells showed no change in the E-cadherin protein levels (S3 Fig), likely due to its high endogenous expression in this cell line. To confirm that this difference between the two cell lines was due to their EMT phenotypes, we repeated the experiment in two additional contrasting cell lines: BT-474 (epithelial-like, high E-cadherin expression, EMT score = -0.548) and MDA-MB-436 (mesenchymal-like, low E-cadherin expression, EMT score = 0.649). Again, FFAR2 overexpression resulted in a pronounced, ligand-mediated increase in E-cadherin expression in the mesenchymal-like cells, but not the epithelial-like cells (S4 Fig).

**Fig 2:**
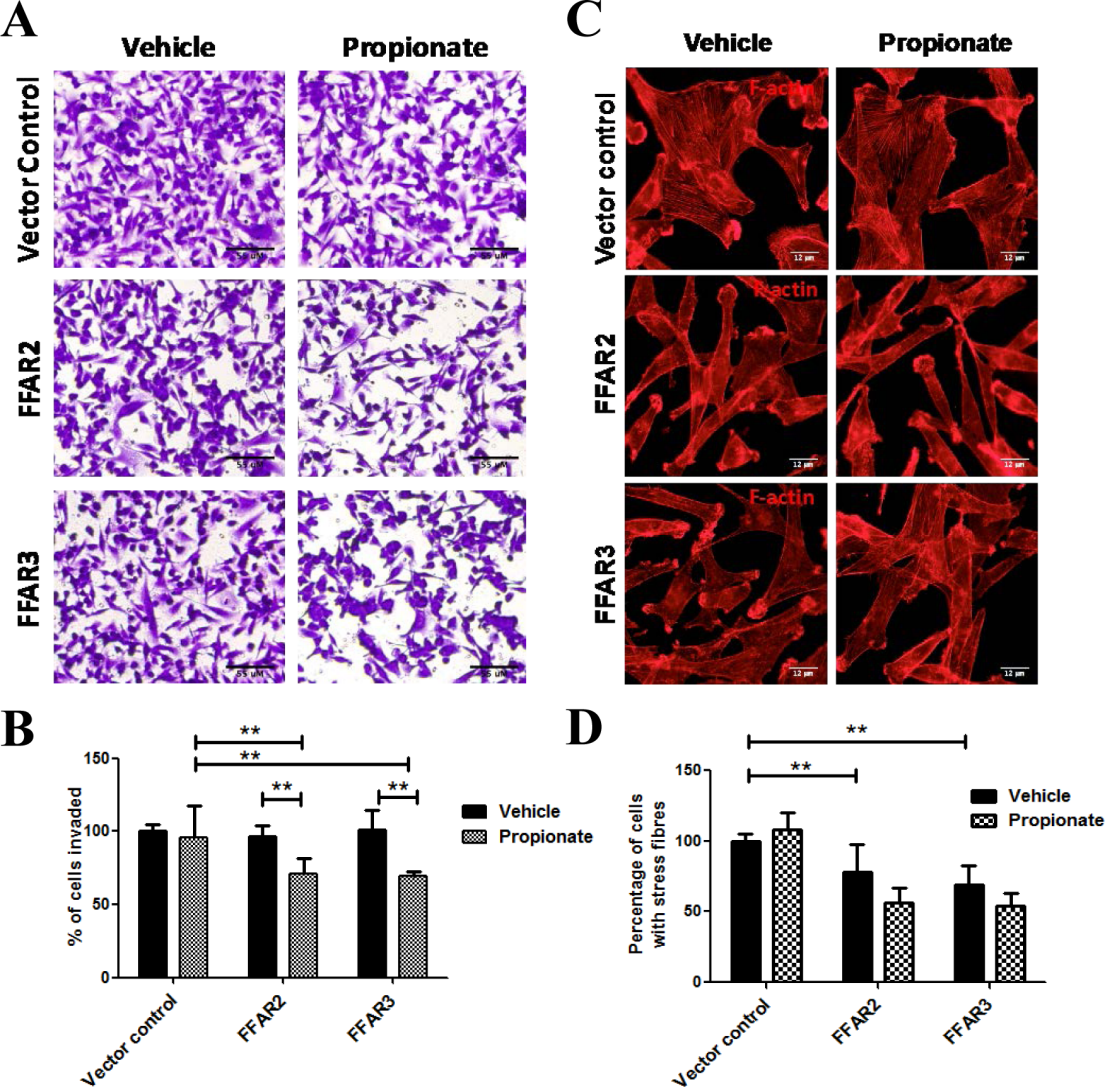
Anti-invasive phenotype in FFAR2- and FFAR3-overexpressing MDA-MD-231 cells. (A) MDA-MB-231 cells were seeded in to the upper chambers of Biocoat Matrigel transwells, then treated with vehicle control or with 1 mM propionate for 24 hours. Representative images show cells that have invaded through the matrix. (B) Quantitation of the invasion assay in (A) shows a significant ligand-mediated reduction in the invasiveness in cells overexpressing FFAR2 and FFAR3, but not vector control cells (N = 3). (C) MDA-MB-231 cells were labeled with rhodamine-phalloidin in order to visualize actin filaments. Representative images are shown. (D) Quantification of cells from (C) that demonstrate the presence of stress fibers (N = 3). (*p< 0.05, **p< 0.01).

To determine whether these changes in E-cadherin expression correlated with changes to the invasive phenotype, cells were treated with propionate for 24 hours and evaluated for their ability to invade a Matrigel-coated matrix. FFAR2- and FFAR3-expressing cells demonstrated a ligand-dependent, significant decrease in invasiveness relative to vector control cells (Fig 2A and 2B). Furthermore, cytoskeletal rearrangement is another notable feature of EMT [45]. Hence, we examined the morphological characteristics of the cytoskeleton by looking at the presence of actin stress fibers. We observed that both FFAR2- and FFAR3-expressing MDA-MB-231 cells showed significantly fewer cells with stress fibers as compared to the control cells (Fig 2C and 2D).

### FFAR2 regulates E-cadherin by inhibition of YAP signaling

**Fig 3:**
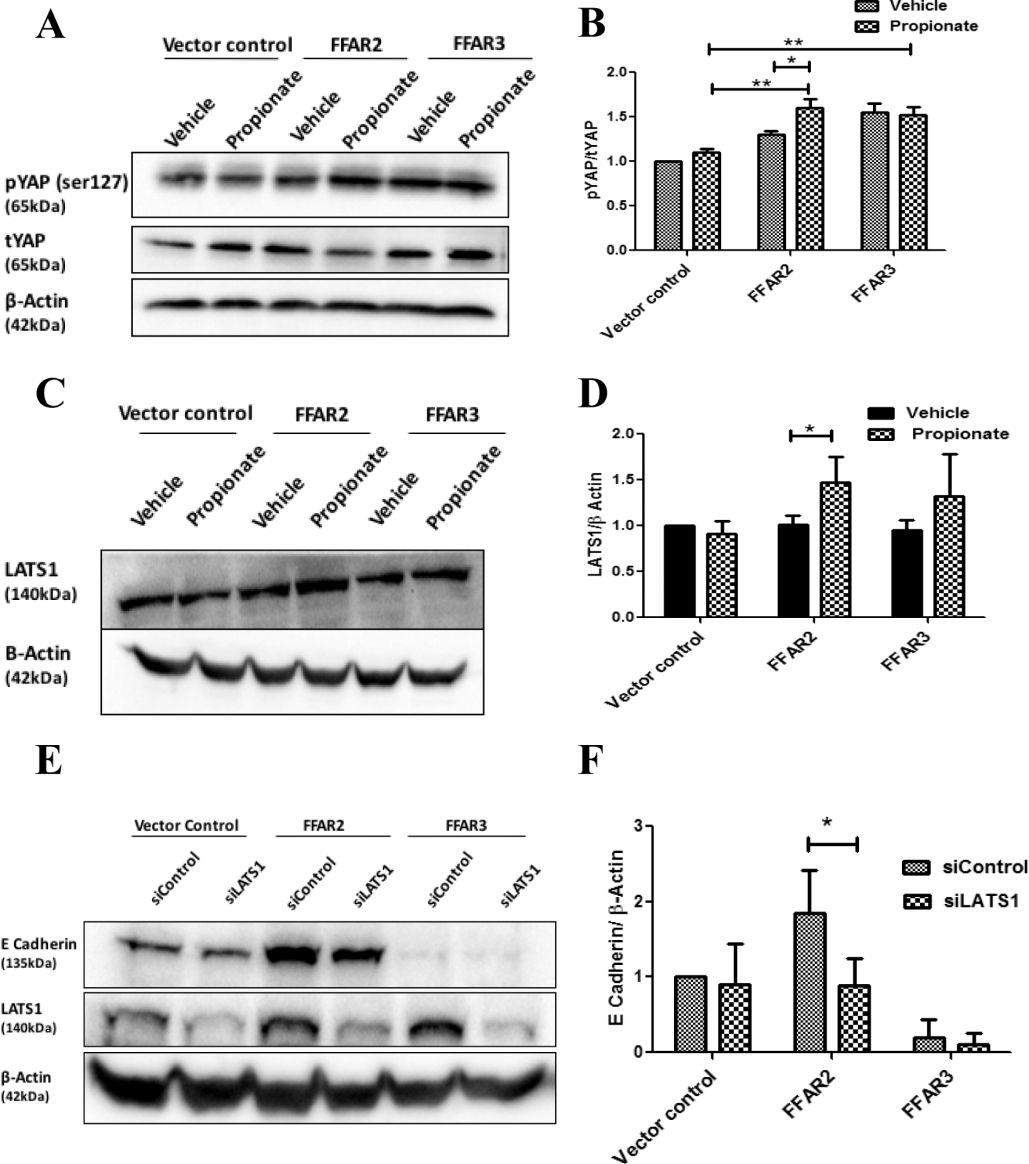
FFAR2 and FFAR3 overexpression regulates Hippo-YAP signaling. (A) MDA-MB-231 cells were treated with vehicle control or with 1 mM propionate for 24 hours, then evaluated for YAP1, pYAP1, and β-actin levels by western blot analysis. Results are representative of three independent experiments. (B) Quantitation of the relative density of the data from (A) demonstrates that pYAP1:YAP1 ratio is increased in both FFAR2- and FFAR3-overexpressing cells, but with a ligand-mediated effect only in FFAR2-expressing cells (N = 3). (C) Cells were treated with vehicle control or with 1 mM propionate for 24 hours, then evaluated for LATS1 and β-actin levels by western blot analysis. Results are representative of three independent experiments. (D) Quantitation of the relative density of the data from (C) demonstrates that FFAR2-overexpressing cells have a ligand-dependent increase in LATS protein (N = 3). (E) Cells were transfected with LATS siRNA or scrambled controls, incubated for 48 hours, then evaluated for E-cadherin, LATS, and β-actin levels by western blot analysis. Results are representative of three independent experiments. (F) Quantitation of the relative density of the data from (E) demonstrates that the FFAR2-mediated increase in E-cadherin can be completely attenuated by knockdown of LATS protein (N = 3). (*p< 0.05, **p< 0.01).

Since the related receptor FFAR1 has been shown to upregulate YAP1 signaling via Gαq and Gαi/o [46], we asked whether FFAR2 and FFAR3 similarly upregulates YAP1 signaling. Both FFAR2- and FFAR3-overexpressing cells showed significant increases in YAP1 phosphorylation (Fig 3A and 3B), consistent with downregulation of YAP1 activity, but interestingly this effect was ligand-dependent only in FFAR2-overexpressing cells. The kinase LATS1 is known to regulate YAP1 activity by phosphorylation at S127 [47]. To determine if the effect of FFAR2 and FFAR3 are mediated by LATS1, we performed a western blot analysis. We observed a significant increase in LATS1 only in FFAR2 cells, suggesting that FFAR2, but not FFAR3, regulates YAP1 *via* modulation of LATS1 expression (Fig 3C and 3D).

To determine whether this pathway is responsible for the FFAR2-mediated increase in E-cadherin expression, we performed siRNA knockdown of LATS1 in our engineered cell lines. This manipulation did not have a significant effect on E-cadherin protein level in control or FFAR3-overexpressing cells, but caused a significant reduction of E-cadherin in FFAR2-overexpressing cells (Fig 3E and 3F), restoring E-cadherin to control levels.

### FFAR3 negatively regulates MAPK signaling

**Fig 4:**
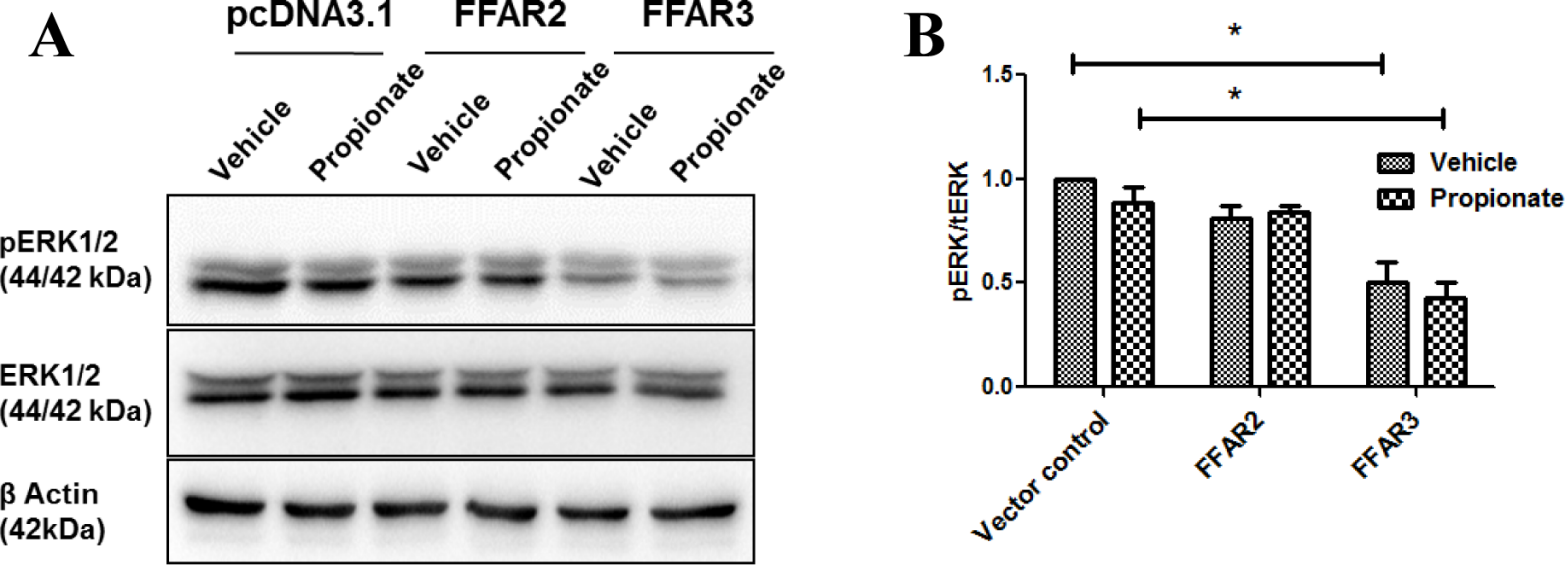
FFAR3 regulates MAP/ERK signaling. (A) MDA-MB-231 cells were treated with vehicle control or with 1 mM propionate for 24 hours, then evaluated for ERK1/2, pERK1/2, and β-actin levels by western blot analysis. Results are representative of three independent experiments. (B) Quantitation of the relative density of the data from (A) demonstrates that pERK1/2:ERK1/2 ratio is significantly reduced in FFAR3-overexpressing cells, but not in FFAR2-overexpressing cells (N = 3).

FFAR2 and FFAR3 have been previously shown to regulate MAPK/ERK1/2 pathway [25] [48, 49]. Therefore, we investigated whether FFAR2 and FFAR3 may be acting through MAPK/ERK. Western blot analysis shows that FFAR3 expressing cells have a significant reduction in ERK phosphorylation, whereas FFAR2 expressing cells remained unchanged relative to control (Fig 4A and 4B).

### Reduced expression of FFAR2 and FFAR3 in invasive and triple negative breast cancer tissues

**Fig 5:**
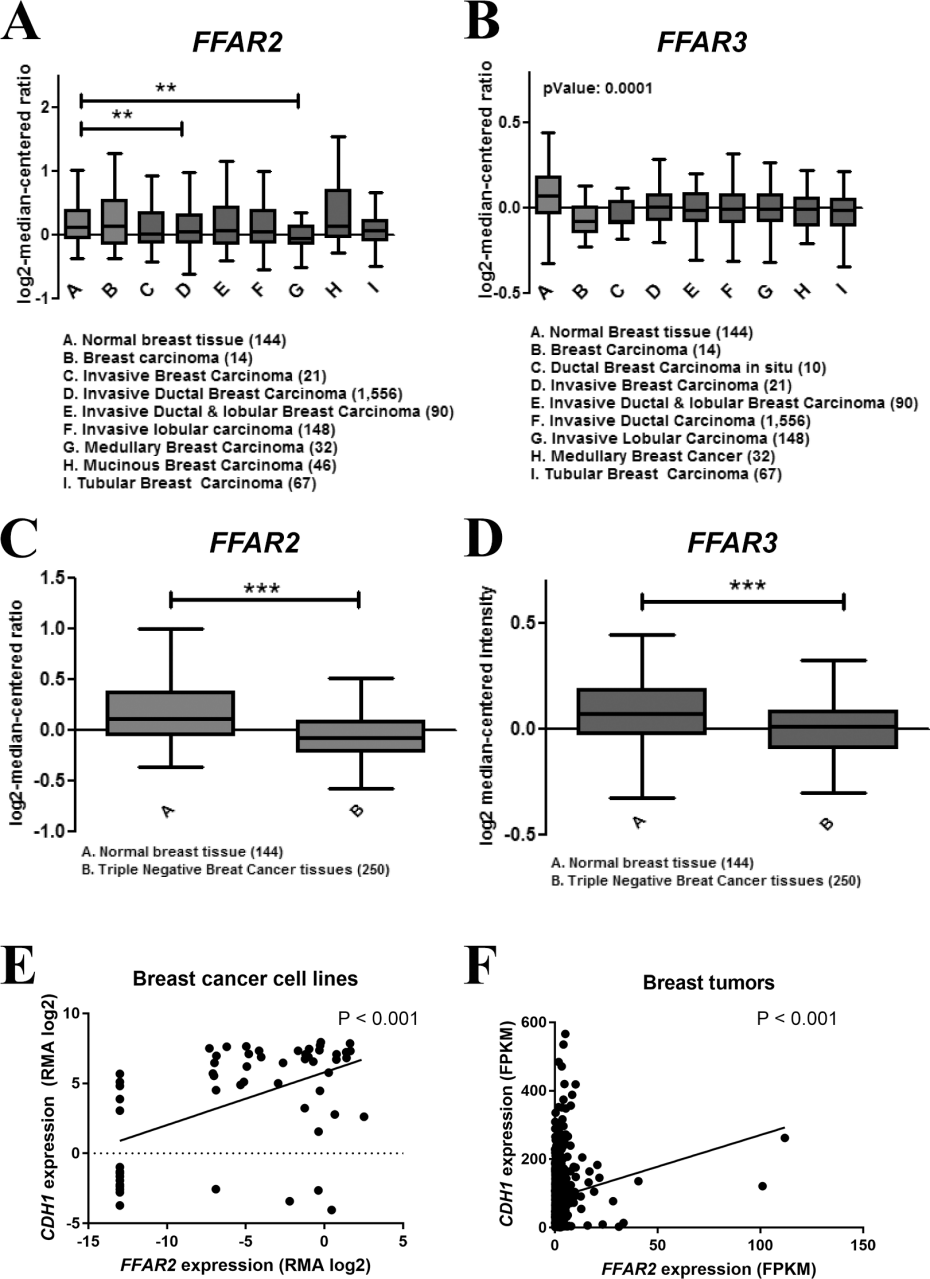
Reduced expression of FFAR2 and FFAR3 in invasive and triple negative breast cancer tissues. The Curtis breast dataset available sourced from Oncomine.org was analyzed for the expression of (A) *FFAR2* and (B) *FFAR3* mRNA in 1992 breast carcinoma and 144 normal breast samples. Whiskers represent the minimum to maximum values. One-way ANNOVA was performed to calculate significance. *FFAR2* expression is reduced in some classes, while *FFAR3* was reduced in all invasive tumor types. The dataset was analyzed for (C) *FFAR2* and (D) *FFAR3* expression level in 144 normal breast tissue samples and in 250 triple negative breast tumors. Expression of both *FFAR2* and *FFAR3* were significantly reduced in triple negative breast tumors. (E) Expression data obtained from the Cancer Cell Line Encyclopedia show that among 57 breast cancer cell lines, there is a significant positive correlation between *FFAR2* and *CDH1* mRNA levels (Pearson r = 0.516, P <0.001). (F) Expression data obtained from the Human Protein Atlas show that among 1075 primary breast tumor samples, there is a significant positive correlation between *FFAR2* and *CDH1* mRNA levels (Pearson r = 0.138, P <0.001).

Since our data suggest that FFAR2 and FFAR3 inhibit EMT, we speculated that expression of these receptors is likely to be reduced in invasive breast cancer tissues. Therefore, to determine the expression pattern of the SCFA receptors in breast tumors, we made use of Curtis breast dataset available on the Oncomine database (www.oncomine.com), which has a gene profile of 1992 breast carcinoma samples and 144 normal breast tissues [50]. Here, both *FFAR2* and *FFAR3* gene expression was significantly downregulated in invasive (Fig 5A and 5B) and triple negative breast cancer tissues (Fig 5C and 5D) when compared to normal breast tissues. Furthermore, to corroborate the involvement of FFAR2 in the regulation of E-cadherin expression, we evaluated the correlation between *FFAR2* and *CDH1* mRNA levels in breast tumors and breast cancer cell lines (Fig 5E and 5F). Expression data for cells lines were obtained from the Cancer Cell Line Encyclopedia (https://portals.broadinstitute.org/ccle) [51]. Expression data for breast tumors were obtained from the Human Protein Atlas (www.proteinatlas.org) [52]. We observed a significant positive correlation between these two genes in both cell lines (Pearson r = 0.516) and breast tumors (Pearson r = 0.138).

## Discussion

The prime goal of this study was to investigate the role of short chain fatty acid receptors (SCFA receptors) in breast cancer metastasis and the mechanism underlying this process. Since metastasis depends in part on the activation of EMT [53], we evaluated effects of FFAR2 and FFAR3 on this process. We observed that FFAR2 resulted in increased *CDH1* (E-cadherin) expression specifically in mesenchymal-like MDA-MB-231 (Fig 1A) and MDA-MB-436 cells (S4 Fig), while no change was observed in epithelial-like MCF-7 (S3 Fig) or BT-474 cells (S4 Fig). In contrast, E-cadherin remained unchanged by FFAR3 overexpression (Fig 1A and S3 Fig). This suggests that FFAR2, but not FFAR3, drives cells toward an epithelial phenotype. The absence of such an FFAR2-mediated effect in MCF-7 and BT-474 cells is likely due to their pre-existing epithelial phenotypes, which are characterized by high endogenous levels of E-cadherin expression [54]. That is, it is unlikely that the contribution of FFAR2 signaling would drive these epithelial cells to adopt further epithelial characteristics.

To confirm that the observed relationship between FFAR2 overexpression and E-cadherin expression was biologically relevant rather than an artefact of *in vitro* manipulation, we evaluated the relative expression patterns of *FFAR2* and *CDH1*. If FFAR2 activity has a functional impact on the regulation of E-cadherin we would expect a positive correlation between these two genes. Indeed, the relationships are positive and highly significant in both cell lines and tumor samples (Fig 5E and 5F). The lower Pearson correlation coefficient observed with the primary tumors (0.138) relative to the cell lines (0.516) is likely the result of the heterogeneity of the tumor samples. Tumor samples contain varying degrees of non-tumor cells, and the net EMT status of the tumor is the result of highly multi-factorial influences. The presence of a positive, and highly significant correlation provides evidence that FFAR2 activity is one of these relevant factors.

The increased E-cadherin expression would be expected to correspond with a reduction in invasive phenotypes. Indeed, we observed a significant decrease in Matrigel invasion and stress fiber formation in FFAR2 expressing MDA-MB-231 cells. Surprisingly, similar phenotypes were also observed in FFAR3 expressing cells (Fig 2C and 2D), despite the fact that E-cadherin level was not changed. This suggested that although FFAR3 disrupts invasion, it is likely to be distinct from EMT. To elucidate alternative mechanisms, we evaluated the MAP/ERK and Hippo-Yap proliferative pathways.

Previous studies demonstrated that both FFAR2 and FFAR3 may be involved in regulating the MAPK-ERK pathway [25, 48, 55], and possibly in the Hippo-YAP pathway [46]. We found that both FFAR2 and FFAR3 increased the phosphorylation of YAP1, but that the effect of FFAR3 was ligand-independent (Fig 3A and 3B). In contrast, we show that FFAR3, but not FFAR2, induces a significant decrease in ERK phosphorylation (Fig 4A and 4B). Since the ERK-MAPK pathway is necessary to regulate transcriptional factors crucial for EMT process [56], it is likely that the FFAR3-mediated downregulation of E-cadherin (Fig 1C) is mediated by inhibition of MAPK signaling.

**Fig 6:**
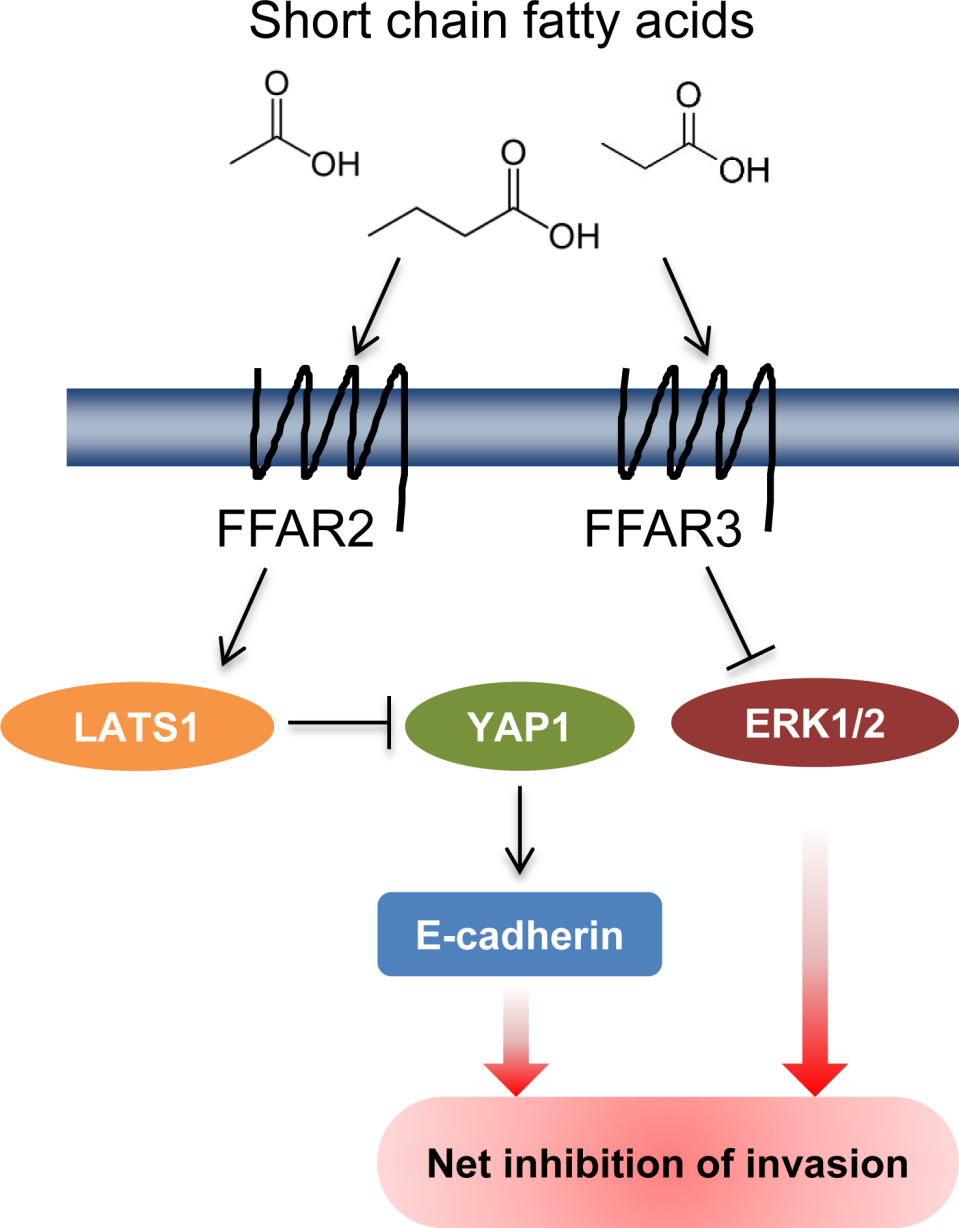
Schematic summarizing the effect of short chain fatty acid receptor overexpression in breast cancer cells. We provide novel data that demonstrate that activation of FFAR2 can activate LATS1 (repressor of YAP1), which results in an increase in E-cadherin levels. In contrast, FFAR3 activity inhibits the MAP/ERK pathway. These processes are likely to underlie the repressive effect of short chain fatty acid receptors on breast cancer cell invasion.

Cumulatively, these results demonstrate that short-chain fatty acids can reduce the invasive potential of breast cancer cells through multiple signaling mechanisms, including the regulation of proliferative pathways, cytoskeletal organization, and expression of adhesion proteins (Fig 6). All of these processes are mediated by cognate receptors FFAR2 and FFAR3, and have the net effect of driving cells from an invasive, mesenchymal phenotype toward a quiescent, epithelial phenotype, thus potentially limiting metastasis.

## Supporting information

S1 Fig Validation of FFAR overexpression cell lines. Engineered MDA-MB-231 cells were evaluated for immunoreactivity with a V5-tag antibody. (A-B) No immunoreactivity was observed in pcDNA3.1-containing cells. (C-D) FFAR2- and (E-F) FFAR3-containing cells demonstrate robust labeling that is largely restricted to the plasma membrane.
(TIF)

S2 Fig Propionate is an effective agonist for FFAR2 and FFAR3. Propionate was prepared by diluting in DMEM to the indicated concentrations and used to treat HEK293 cells transfected with pcDNA3.1 or the indicated FFAR. Evaluation of TGFα shedding demonstrates that propionate has EC_50_ values of 7 and 10 μM for FFAR2 and FFAR3, respectively.
(TIF)

S3 Fig Short chain fatty acid receptor signaling does not affect E-cadherin expression in MCF7 cells. (A) MCF7 cells were treated with vehicle control or with 100μM propionate for 24 hours, then evaluated for E-cadherin levels by western blot analysis. Results are representative of 3 independent experiments. (B) Quantitation of the relative density of the data from (A). No significant change in E-cadherin was observed (N = 3).
(TIF)

S4 Fig Transient over-expression of FFAR2 causes a ligand-dependent increase in E-cadherin expression in mesenchymal cells, but not epithelial cells. Mesenchymal-like MDA-MB-436 cells and epithelial-like BT-474 cells were transiently transfected with pcDNA3.1 (vector) or FFAR2-expressing plasmids. After 48 hours, cells were treated with vehicle or 1 mM propionate. 24 hours after treatment, cell lysates were collected and evaluated for E-cadherin and β-actin proteins by western analysis. Increased E-cadherin expression was observed in MDA-MB-436 cells, but not BT-474 cells. Images are representative of 2 independent experiments.
(TIF)
