# Supplementary figures and images for: Short-chain fatty acid receptors inhibit invasive phenotypes in breast cancer cells
Source: PLoS One. 2017 Oct 19;12(10):e0186334. doi: 10.1371/journal.pone.0186334 (PMC5648159; doi:10.1371/journal.pone.0186334)

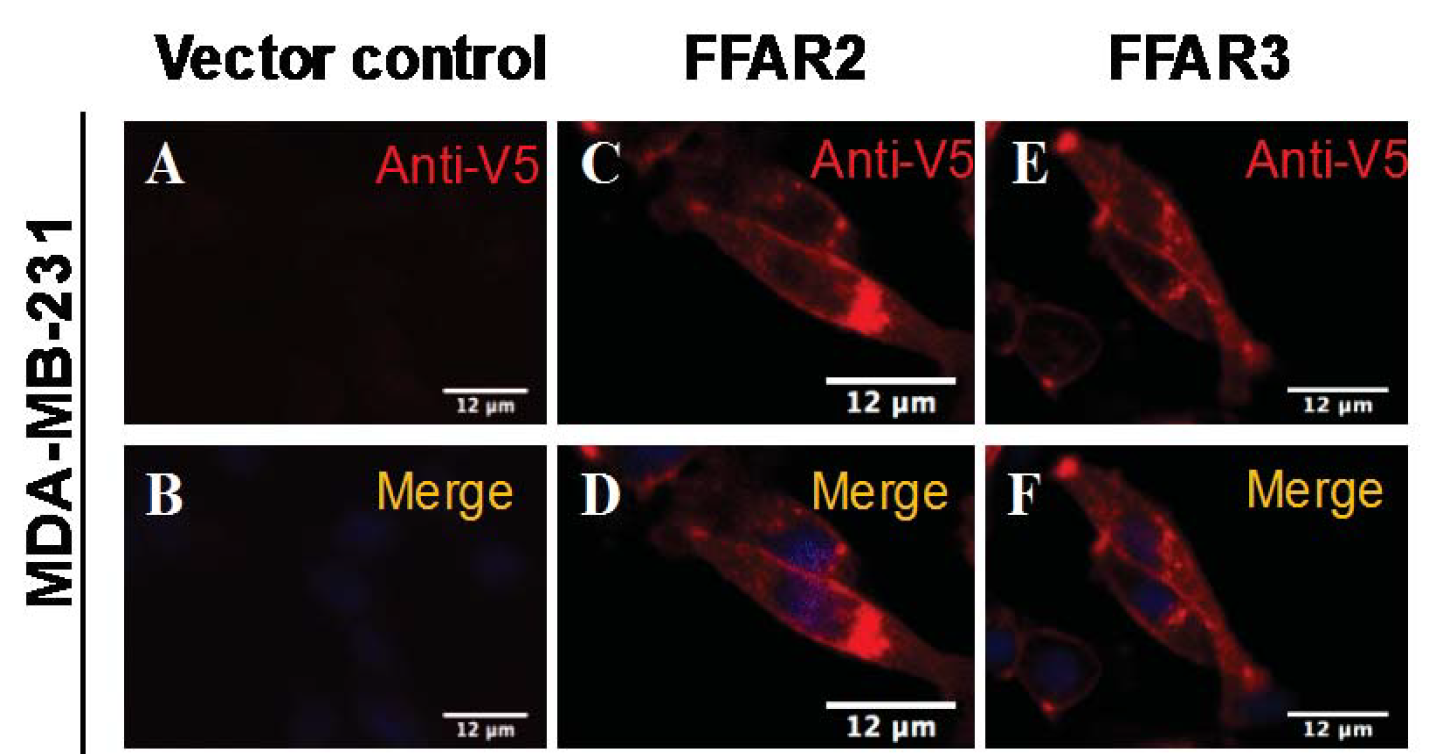

Supplement: S1 Fig — Engineered MDA-MB-231 cells were evaluated for immunoreactivity with a V5-tag antibody. (A-B) No immunoreactivity was observed in pcDNA3.1-containing cells. (C-D) FFAR2- and (E-F) FFAR3-containing cells demonstrate robust labeling that is largely restricted to the plasma membrane. (TIF) [file pone.0186334.s001.tif]

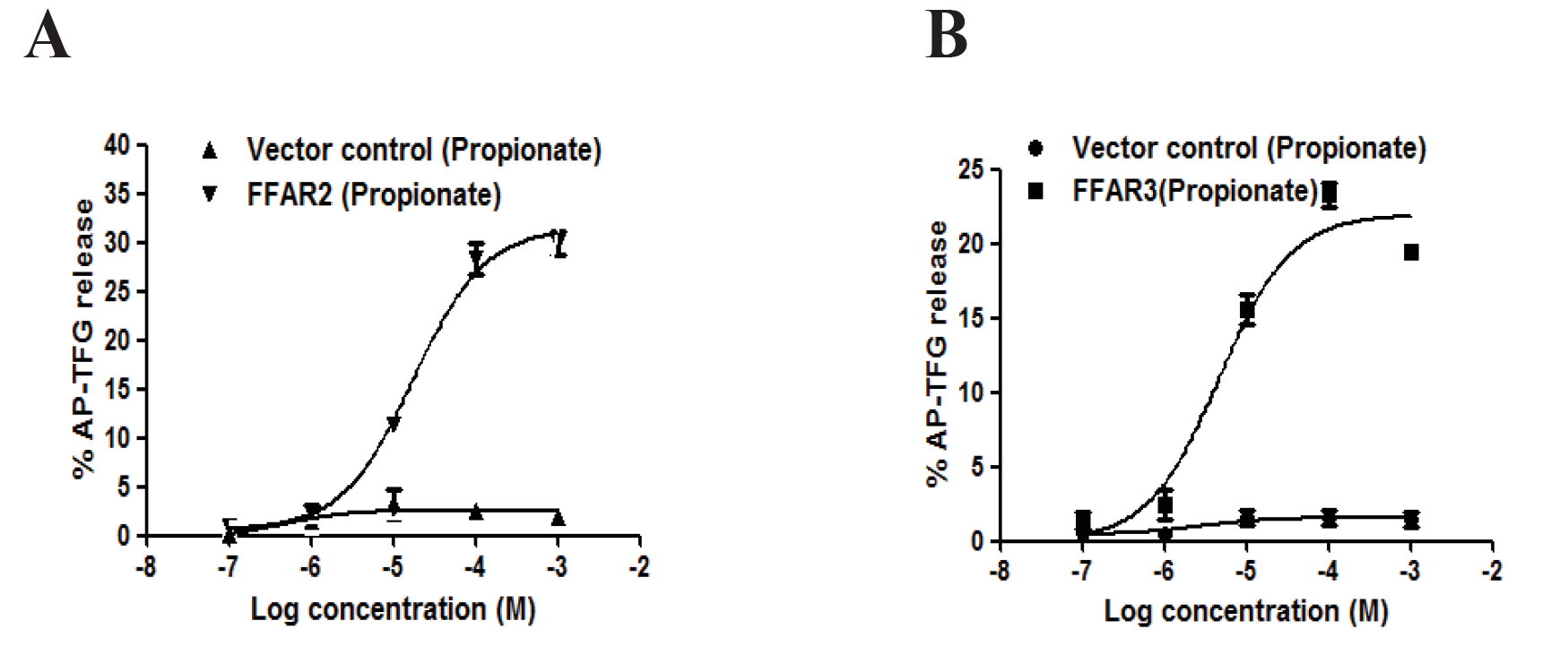

Supplement: S2 Fig — Propionate was prepared by diluting in DMEM to the indicated concentrations and used to treat HEK293 cells transfected with pcDNA3.1 or the indicated FFAR. Evaluation of TGFα shedding demonstrates that propionate has EC50 values of 7 and 10 μM for FFAR2 and FFAR3, respectively. (TIF) [file pone.0186334.s002.tif]

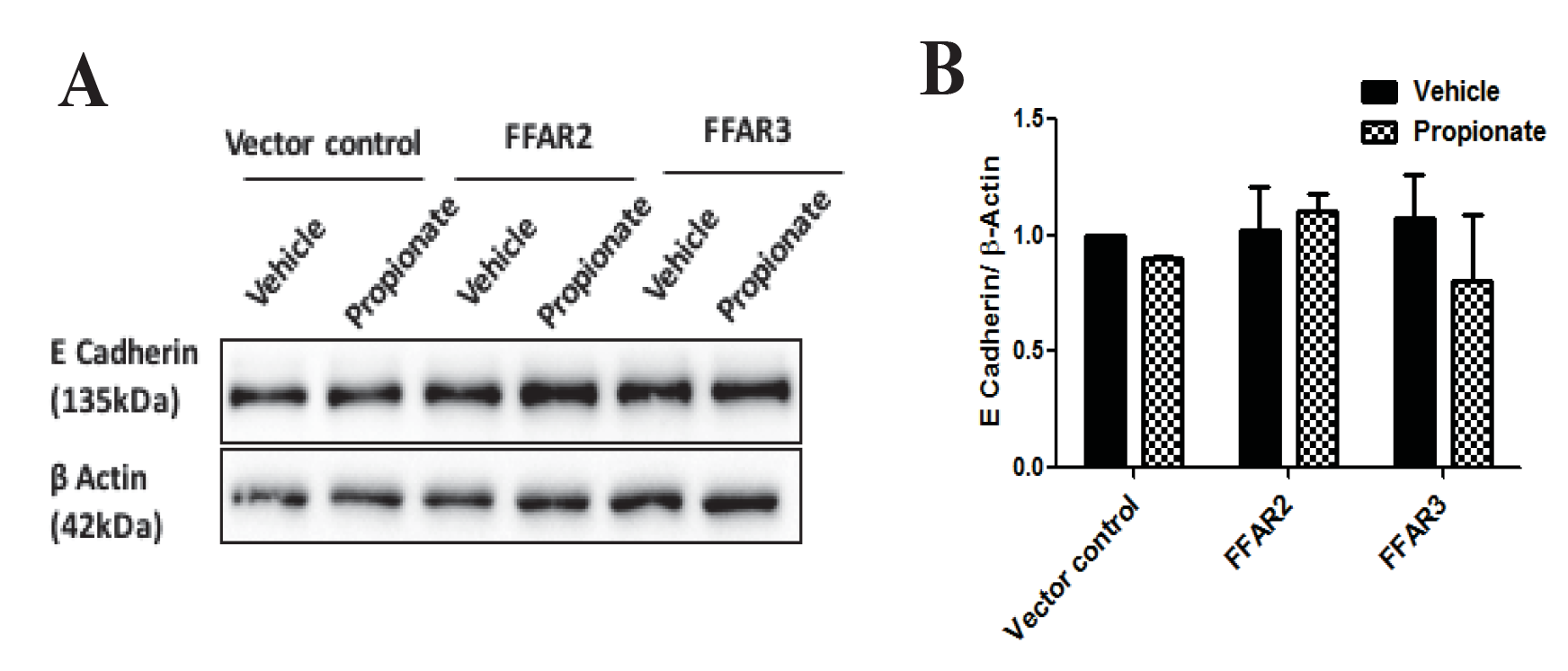

Supplement: S3 Fig — (A) MCF7 cells were treated with vehicle control or with 100μM propionate for 24 hours, then evaluated for E-cadherin levels by western blot analysis. Results are representative of 3 independent experiments. (B) Quantitation of the relative density of the data from (A). No significant change in E-cadherin was observed (N = 3). (TIF) [file pone.0186334.s003.tif]

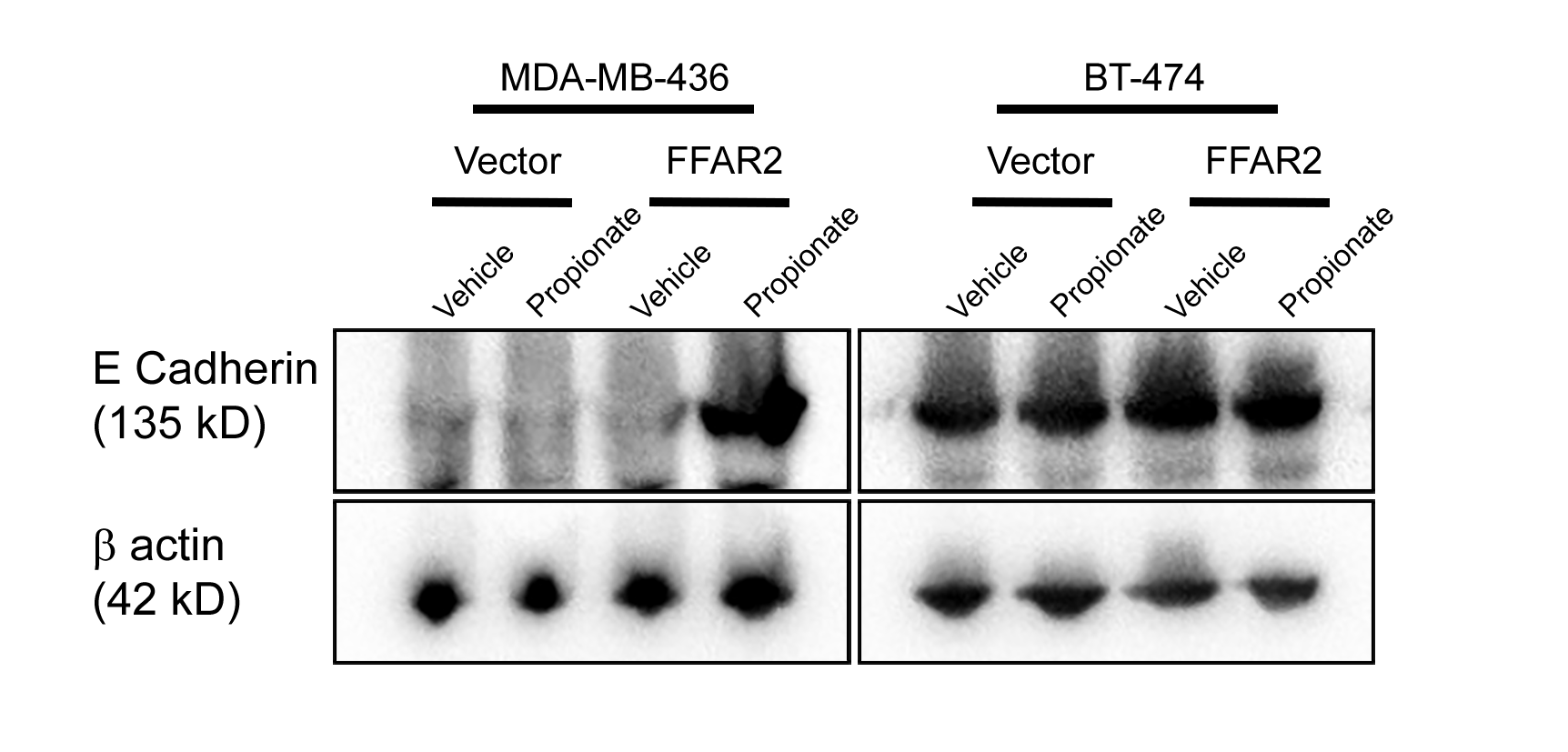

Supplement: S4 Fig — Mesenchymal-like MDA-MB-436 cells and epithelial-like BT-474 cells were transiently transfected with pcDNA3.1 (vector) or FFAR2-expressing plasmids. After 48 hours, cells were treated with vehicle or 1 mM propionate. 24 hours after treatment, cell lysates were collected and evaluated for E-cadherin and β-actin proteins by western analysis. Increased E-cadherin expression was observed in MDA-MB-436 cells, but not BT-474 cells. Images are representative of 2 independent experiments. (TIF) [file pone.0186334.s004.tif]
